# m6A Regulator-Mediated Methylation Modification Patterns and Tumor Microenvironment Infiltration Characterization in Acute Myeloid Leukemia

**DOI:** 10.3389/fimmu.2021.789914

**Published:** 2021-11-23

**Authors:** Ashuai Du, Xin Wu, Yunmei Gao, Baili Jiang, Jianlong Wang, Pan Zhang, Qiangqiang Zhao

**Affiliations:** ^1^ Department of Infectious Diseases, Third Xiangya Hospital, Central South University, Changsha, China; ^2^ Department of Infectious Diseases, Guizhou Provincial People’s Hospital, Guizhou, China; ^3^ Department of Orthopedics, Third Xiangya Hospital, Central South University, Changsha, China; ^4^ Department of Hematology, The Qinghai Provincial People’s Hospital, Xining, China; ^5^ Department of Medical Oncology, The Xiayi County Traditional Chinese Medicine Hospital, Shangqiu, China; ^6^ Department of Blood Transfusion, The Third Xiangya Hospital, Central South University, Changsha, China

**Keywords:** m6A, tumor microenvironment, AML, immunotherapy, mutation burden

## Abstract

Recent studies have demonstrated epigenetic regulation of immune responses. Nevertheless, the underlying effect of RNA N6-methyladenosine (m6A) modifications on tumor microenvironment cell infiltration remains elusive. In this study, we thoroughly assessed m6A modification patterns of 255 myeloid leukemia specimens based on 23 m6A regulators. Consensus clustering of the 23 m6A regulators was performed to determine three distinct m6A modification patterns that were remarkably consistent with three immunophenotypes of tumors: immunorejection, immune activation, and immune inertness. Further evaluation and prognostic analysis of the m6A modification patterns of individual tumors revealed that low m6A score was characterized by increased mutational burden, immune activation, and survival rates, whereas high m6A score was characterized by poorer survival rates and the absence of effective immune infiltration. In addition, this study investigated the association between m6A regulators and antitumor immune responses and discovered higher expression of the immune regulators *PD-L1*, *PD-L2*, *MRP1*, and *MRP2* in low m6A scores. Generally, the expression pattern of m6A regulators was remarkably associated with prognostic results and antitumor immune responses in acute myeloid leukemia and may be an underlying target and biological marker for immune therapies.

## Introduction

Acute myeloid leukemia (AML) is one of the most common and invasive hematological carcinomas in adults, accounting for approximately 1% of all cancers (1, 2). AML is characterized by the culmination of immature bone marrow hematopoietic cells, particularly in the bone marrow. Peripheral blood participation is also common and may cause malignant infiltration into the skin, lymph nodes, spleen, liver, and CNS (3). The primary treatment strategies for AML, namely, intensive induction chemical therapy and postremission treatment, have remained largely unchanged over the last 30 years, and patient survival has not substantially improved (4, 5). Despite the fact that substantial studies have assisted in revealing the genomic picture of AML and better comprehending its evolution, converting such knowledge into better therapeutic approaches has just begun. Therefore, the determination of underlying markers will improve the diagnostic, therapeutic, and prognostic results of patients with acute myeloid leukemia.

In recent years, much attention has been paid on the effects of tumor microenvironments (TME) on the progression of cancer (6). As a result, alterations in TME components have been identified in almost all cancer types at every stage of malignant development, which can assist in understanding tumor development and identifying underlying treatment targets (7). For example, different TME factors, such as dissolvable factors, inhibitory immunocytes, and alterations in the ECM, have been shown to jointly act to suppress tumor immune therapy, trigger chemotherapy resistance, and facilitate mammary carcinoma progression (8). Similarly, the breakthrough discoveries that led to the present *PD-1*/*PD-L1*-targeted immune treatment were derived from evaluating tumor-stromal mutual effects and particular variations within the TME (9). The TME has been found to be a key determining factor for diagnostic and therapeutic reactions in tumor patients (10, 11). The complexity of the TME is demonstrated by the multiple interactions between tumor, stromal, immune, and mesenchymal cells, *via* various dissolvable factors and variations in ECM components (12). As two primary nontumor cell colonies in the TME, stromal cells and infiltration immunocytes have been implicated in the diagnostic and prognostic outcomes of tumors. TME is considered a consensus area for the identification of novel tumor biomarkers (13, 14). For that reason, by comprehensively analyzing the inhomogeneity and intricacy of TME, it may be possible to determine different tumor immune phenotypes and the ability to direct and forecast immunotherapy responsiveness could be improved. Furthermore, potential biomarkers could be identified, which would be useful in predicting patient responses to immune therapy and facilitate the discovery of novel treatment targets (15, 16).

N6-methyladenosine (m6A), which is a methylation at N6 of adenosine, has been discovered to be crucial for the regulation of RNA transcription, processing, and translational and metabolic activities (17, 18). The biological functions of these m6A modifications are kinetically modulated by a methyltransferase complex consisting of RNA methyl transferases (“writers”), demethylases (“erasers”), and m6A-binding proteins (“readers”). m6A methylation is performed by *RBM15*, *ZC3H13*, *METTL3*, *METTL14*, *WTAP*, and *KIAA1429*, whereas removal of the methylation is performed by demethylases *FTO* and *ALKBH5*. Moreover, a group of particular RNA-binding proteins, including *YTHDF1/2/3*, *YTHDC1/2*, *HNRNPA2B1*, *LRPPRC*, and *FMR1*, can recognize m6A patterns and hence affect m6A function (19, 20). Studies have found that m6A modulators are crucial for various biological functions *in vivo*, which participate in the development of a variety of cancers (21, 22), including bladder cancer (23), acute myeloid leukemia (24), glioblastoma (25), and hepatocellular carcinoma (26). Increasing evidence suggests that dysregulated expression and genetic changes in m6A modulators are related to the dysregulation of a number of biological activities, such as aberrant regulation of cell death and proliferation, developmental defects, malignant tumor development, damaged self-recovery ability, and aberrant immunoregulation (27, 28).

However, due to technology restrictions, previous studies have been restricted to one or two m6A modulators and cytotypes, whereas antitumor effects are characterized by many tumor suppressors interacting in remarkable coordination. Thus, a thorough understanding of TME infiltrative cell characteristics under the mediation of several m6A modulators would help to better understand TME immunoregulation. In this study, we combined the genome data from 255 AML specimens to thoroughly assess the m6A modification profile and correlate m6A modification patterns with TME infiltrative cell features. We revealed three different m6A modification patterns and interestingly discovered that TME features in these three patterns remarkably coincided with immunorejection, immune activation, and immunodeficiency phenotypes, respectively, revealing that m6A modifications have a nonnegligible effect on the formation of different TME features. To this end, a scoring system was developed to quantify the pattern of m6A modification in a single patient.

## Methods

### AML Dataset Sourcing and Preprocessing

The process of the present study is presented in **Supplementary Figure S1**. Public genetic expression information and complete clinical annotations were retrieved from the GEO, TCGA, and GTEx databases. Patients with no survival data were excluded. Data from the eligible GSE71014 GEO dataset were used for deeper assays. For the microarray performed using this dataset, we acquired the normalized matrix data directly through download. For the TCGA dataset, RNA sequencing data of gene expression were acquired from the GDC *via* the R Package-Age TCGA biolinks (29), which was developed particularly for comprehensive assays of GDC data. Somatic mutation information was obtained from the TCGA database. Datasets were obtained for copy number variant (CNV) assays. The results were assayed using R 3.6.1 and R Bioconductor software.

### Unsupervised Clustering for 21 m6A Regulators

Twenty-three modulators from the integrated GEO dataset were extracted to identify the different m6A modifications mediated by m6A regulators. These 23 m6A modulators included eight writers (*METTL3*, *METTL14*, *METTL16*, *WTAP*, *VIRMA*, *ZC3H13*, *RBM15*, *RBM15B*), two erasers (*ALKBH5* and *FTO*), and 11 readers (*YTHDC1*, *YTHDC2*, *YTHDF1*, *YTHDF2*, *YTHDF3*, *HNRNPC*, *FMR1*, *LRPPRC*, *HNRNPA2B1*, *IGFBP1*, *IGFBP2*, *IGFBP3*, *RBMX*). Based on the expression of 23 m6A regulators, an unsupervised cluster assay was employed to determine different m6A modification patterns and to categorize patients for more in-depth assays. ConsensusClusterPlus software was employed to implement the aforementioned procedures.

### Estimation of TME Cellular Infiltration

ssGSEA algorithms were used to quantify the comparative abundance of each infiltrative cell activity within the TME. The gene markers for each TME infiltrative immune cytotype were acquired from the research of Charoentong, which identified a variety of human immunocyte subgroups such as stimulated CD8 T cells, stimulated dendritic cells, macrophages, and NKT and modulatory T cells (30). The enrichment values computed *via* ssGSEA assay were used to calculate the comparative abundance of each TME infiltrative cell in each specimen.

### Determination of Differently Expressed Genes Between Distinct m6A Phenotypes

To identify the m6A-associated genes, the patients were divided into three different m6A modification pattern groups based on their expression of the 23 m6A modulators. The experiential Bayesian method of the limma R software was employed to identify differently expressed genes (DEGs) between different modification patterns (31). Statistical significance was set at *p* < 0.001 for identifying DEGs. Functional enrichment analysis of DEGs was performed using clusterProfiler (32) R software to determine GO classes, such as BP, MF, and CC. A pathway enrichment assay using the KEGG database was also completed using this software. Statistical significance was set at *p* < 0.05.

### Generation of m6A Genetic Signature

To quantify the m6A modification pattern of a single tumor, we established a scoring system to assess the m6A modification features of each patient with AML, i.e., the m6A genetic signature. The DEGs detected in distinct m6A clusters were normalized, and overlapping genes were extracted. Patients were divided into groups for more in-depth analysis using unsupervised clustering methods to analyze the overlapping DEGs. Consensus clustering algorithms were used to define the quantity of genetic clusters and the relevant steadiness. We then completed a prognosis assay for each gene in the signature using a univariable Cox regression pattern. Genes with remarkable prognostic outcomes were extracted for deeper assays. Our team subsequently performed PCA to establish m6A-associated genetic signatures.

### Validation of mRNA Expression of Prognostic Genes Between AML and Healthy Samples by qRT-PCR

Plasma samples from 10 patients with AML and 10 healthy controls were collected from the People’s Hospital of Guizhou Province. Ethical approval was granted by the Human Research Ethical Board of GPPH. The mRNA expression levels of the four immune genes in the specimens were determined *via* qRT-PCR. Overall RNA from AML and normal control plasma specimens was prepared using Trizol reagent (Servicebio, Wuhan, China) as per the specification. RNA was then converted to cDNA by reverse transcription using the RevertAid First-Strand cDNA Preparation Kit (Thermo Fisher Scientific, Waltham, MA, USA). The genetic expression was normalized to that of GAPDH. FastStart Universal SYBR Green Master (Roche) was used to quantify the real-time PCR assay, using StepOne (Applied Biosystems, Waltham, MA, USA). The primer sequences are presented in **Supplementary Table S1**. Each RNA specimen was prepared three times. To compare the expression levels of the different specimens, the comparative expression of genes associated with inflammatory responses was calculated using the 2^−ΔΔCt^ method.

### Immunohistochemistry and Immunofluorescence

The sections were dewaxed two times with xylene and gradients of different concentrations of anhydrous ethanol (100%, 95%, 90%, 80%, and 70% alcohol), placed into citrate buffer solution (0.01 mol/L, pH 6.0), and then boiled for 3 min in an autoclave. The sections were then sealed with 5% BSA and placed in an incubator at 37°C for 0.5 h. The slices were cultivated with the first antisubstance at 4°C nightlong. The sections were cultivated with a second antibody (horseradish peroxidase-labeled) for 60 min at room temperature and stained with DAB. Eventually, the sections were restained with hematoxylin, desiccated, and then fixed. The expression of *PD-L1*, *PD-L2*, *MRP1*, and *MRP2* proteins in bone marrow tissue was detected by immunofluorescence. In the paraffin-embedded bone marrow pathology sections, the aforementioned identification process was completed according to the specifications provided by the supplier. The nuclei were stained with DAPI, and the relevant images were examined using an inverted fluorescence microscope.

### Statistical Analysis

One-way ANOVA and Kruskal-Wallis tests were used to compare the diversities among ≥3 groups (33). The cutoff points for each dataset subgroup were identified *via* the Survminer R package based on the association between m6A score and patient survival. Applying the “surv-cutpoint” function, all potential cutoff points were iteratively tested to identify the maximal rank statistic to dichotomize the m6A score. Patients were then separated into high and low m6A score groups based on the maximum chosen log rank statistic to reduce the batch effect of the computation. Survival curves for progression assays were produced using the KM approach, and log-rank tests were used to analyze the significance of differences. The waterfall function of the maftools package was employed to present the mutations in patients in the high and low m6A score groups from the TCGA-LAML cohort. The R package from RCircos was used to map CNVs in the 23 m6A modulators. All statistical *p* results were bilateral, and *p* < 0.05 was considered statistically significant. The entire processing was completed using R3.6.1 software.

## Results

### Landscape of Gene Variation of m6A Regulators in AML

**Figure 1:**
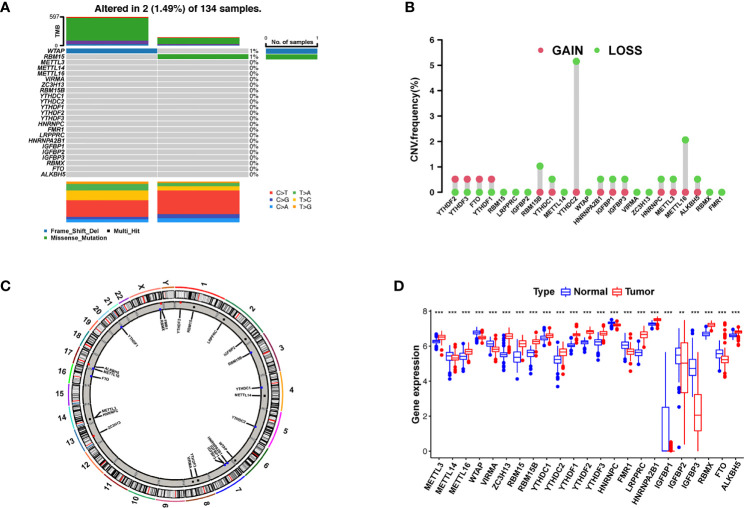
Landscape of gene and expression changes of m6A modulators in AML. **(A)** The mutational frequency of 23 m6A modulators in 134 patients with AML from the TCGA-AML cohort. Each column reflects a single patient. The bar plot above presents TMB. The figure on the right reflects the mutational frequency of each modulator. The bar plot on the right presents the level of each variation type. **(B)** The copy number variant (CNV) change frequency of the m6A regulators from the GSE71014 cohort. The column height reflects the change frequency. Deletion frequency = green point; magnification frequency = red point. **(C)** The site of CNV change of the m6A regulators on 23 chromosomes from the GSE71014 cohort. **(D)** The expression of 23 m6A regulators between healthy and tumor samples. Cancer = red; healthy = blue. The upper and lower ends of the boxes reflect the quartile deviation of the data. The lines in the boxes reflect the midvalue, and the black points denote outliers. The asterisks reflect the statistical *p* result (^**^ *p* < 0.01; ^***^ *p* < 0.001).

We first summarized the incidence of CNVs and somatic mutations in 23 m6A regulators in AML, including eight writers, two erasers, and 13 readers (**Supplementary Table S2**). Mutations in the m6A regulators occurred in two of the 134 samples, with a frequency of 1.49%. The assay of AML specimens found that only *WTAP* and *RBM15* showed any mutation frequency, while the other genes did not (**Figure 1A**). Wild-type *WTAP* was more significantly expressed (**Supplementary Figure S2**).

Investigation of CNVs showed prevalent CNV alterations in the 23 regulators, most of which were deletions in copy number, with *RBM15B*, *YTHDC2*, and *METTL16* frequently demonstrating CNV deletion (**Figure 1B**). The site of CNV variation in the m6A regulators on the chromosome is shown in **Figure 1C**. To determine if these gene variants affect the expression of m6A modulators in patients with leukemia, we investigated the expression levels of regulator mRNA in healthy and tumor specimens and discovered that CNVs may be a major factor contributing to the disruption of m6A regulator expression. The expression of CNV-amplified m6A regulators *YTHDF1*, *YTHDF2*, *YTHDF3*, and *FTO* was remarkably higher in AML specimens than in healthy specimens (**Figure 1D**). Survival analysis of high and low m6A regulator expression is shown in **Supplementary Figure S3**. The survival analysis demonstrates a remarkable level of inhomogeneity in the gene and expression of m6A modulators in healthy and AML samples, suggesting that imbalances in m6A regulator expression play a key role in the development of AML.

### m6A Methylation Modification Patterns Mediated by Regulators

**Figure 2:**
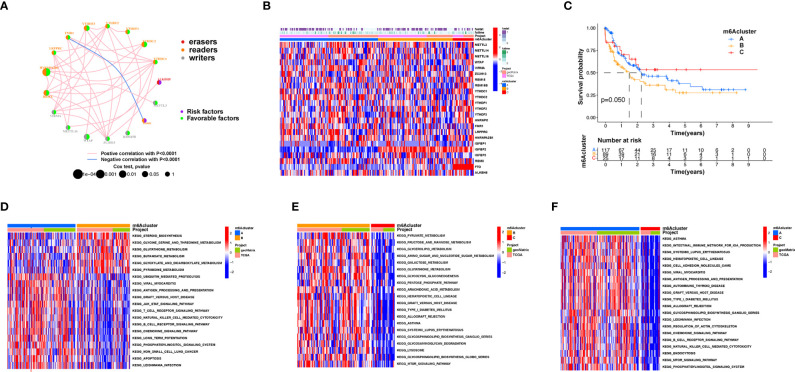
Patterns of m6A methylation modification and biological features of each pattern. **(A)** Mutual effects of m6A modifiers in AML. The circle size represents the role of every modulator in prognostic results, and the data computed *via* log-rank test ranged from *p* < 0.001, *p* < 0.01, *p* < 0.05, and *p* < 0.1, respectively. Purple points in the circles = prognostic risk factors; green in the circles = prognostic protection factors. The line linking the moderators indicates their interaction. Negative association is presented in blue whereas positive association is presented in red. **(B)** Unsupervised cluster of 23 m6A modulators in the AML cohort. The m6A cluster was utilized as patient annotations. Red represents high expression of modulators whereas blue represents low expression. **(C)** Survival assay for the three m6A modification patients based on 231 patients with AML, including 117 patients in m6A cluster A, 89 patients in m6A cluster B, and 25 patients in m6A cluster C. KM curves (log-rank *p* score < 0.05) displayed remarkable survival diversity among the three m6A modification patterns. m6A cluster C presented a remarkably improved overall survival in contrast to the other m6A clusters. **(D–F)** KEGG enrichment assay displaying the stimulation status of biological pathways of each m6A modification pattern. Red represents stimulated pathways whereas blue represents suppressed pathways. The AML cohorts were utilized to annotate specimens. **(D)** m6A cluster A versus m6A cluster B; **(E)** m6A cluster B versus m6A cluster C; **(F)** m6A cluster A vs. m6A cluster C.

An overview of m6A modulator mutual effects, modulator association, and prognosis of AML patients is described *via* the m6A modulator net (**Figure 2A**). The R software of Consensus Cluster Plus was employed to categorize patients with distinct m6A modification patterns *via* quantification of the expression of the 23 m6A modulators, and three different modification patterns were ultimately determined *via* unsupervised clustering (**Figure 2B**), including 117 patients with pattern A, 89 patients with pattern B and 25 patients with pattern C. We named these patterns m6A clusters A–C, respectively (**Supplementary Table S3**). The prognosis assay for the three primary m6A modification subgroups revealed specific survival benefits for the m6A cluster C modification pattern (**Figure 2C**). To explore the biological activities of the different m6A modification patterns, a KEGG enrichment assay was performed, and it was found that the significant enrichment of m6A cluster A in stromal and carcinogenesis stimulation pathways such as T-cell receptor, B-cell receptor, and JAK-STAT signaling pathways, cell apoptosis, and NK-mediated cell toxicity (**Figure 2D**). m6A cluster B displayed enrichment of pathways related to pyruvate, fructose, mannose, glycerolipid, and galactose metabolic activities, as well as glycosaminoglycan degradation (**Figure 2E**). m6A cluster C was significantly associated with B-cell receptor, chemokine, cell attachment, MTOR signaling pathways, and immune suppression biological processes (**Figure 2F**). We found that the three m6A modification patterns exhibited distinctly different TME infiltrative cell profiles. Cluster A was categorized as an immunorejection phenotype characterized by congenital immunocyte infiltrative activity and stromal stimulation. Cluster B was categorized as immune metabolism, regulating immune activation through the metabolic programs of different cell subpopulations, and cluster C was characterized by immunosuppression.

### TME Infiltrative Cell Patterns in Distinct m6A Modification Patterns

**Figure 3:**
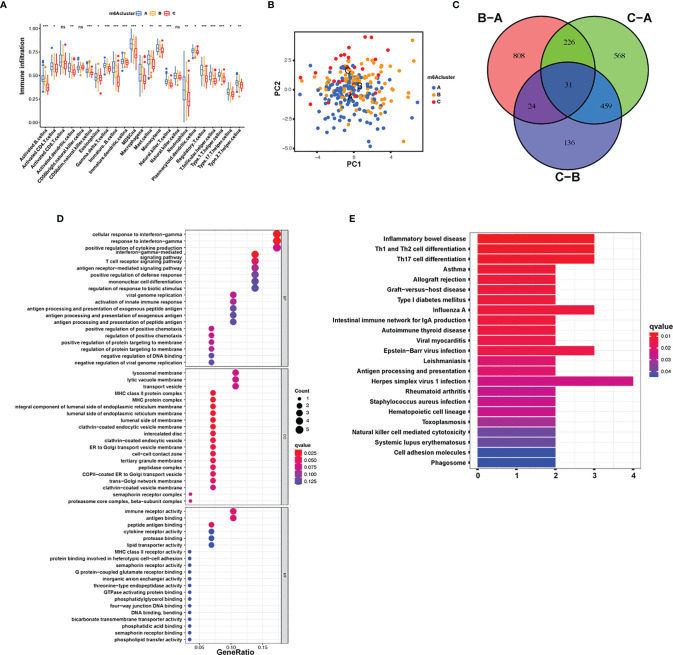
TME cellular infiltration and transcription of different m6A modification patterns. **(A)** The abundance of each TME infiltration cell type in the three m6A modification patterns. The upper and lower ends of the boxes represent the quartile deviation of the data. The lines in the boxes represent the midvalue, and the black points represent the outliers. The asterisks denote the statistical *p*-value (^*^ *p* < 0.05; ^**^ *p* < 0.01; ^***^ *p* < 0.001; ns, non-significant). **(B)** PCA for the transcriptomic profiles of the three m6A modification patterns, displaying evident diversity in the transcription between different modification patterns. **(C)** The 31 m6A phenotype-associated genes presented in a Venn illustration. **(D, E)** Functional annotation of m6A-associated genes *via* GO and KEGG enrichment assays. The color depth of the barplots denotes the quantity of enriched genes.

The following assay of TME infiltrative cell results revealed that m6A cluster A had significant enrichment of congenital immunocyte infiltrative activity, including NK, macrophages, eosinophils, mastocytes, MDSCs, and PDCs (**Figure 3A**). However, significant differences occurred in the m6A transcriptional profile among the three distinct m6A modification patterns (**Figure 3B**). To explore the underlying biological behavior of each m6A modification pattern, the limma package was used to identify 31 DEGs associated with m6A phenotypes (**Figure 3C** and **Supplementary Table S4**). Cluster Profiler software was employed to complete the GO enrichment assay of the DEGs (**Supplementary Table S5**). The biological processes with significant enrichment were denoted as KEGG pathways (**Supplementary Table S6**). Interestingly, the genes that were enriched were significantly associated with m6A modification and immune activity, which confirms that m6A modification has a nonnegligible effect on the immunoregulation of the TME (**Figures 3D, E**).

### m6A Methylation Modification Patterns and Functional Annotation

**Figure 4:**
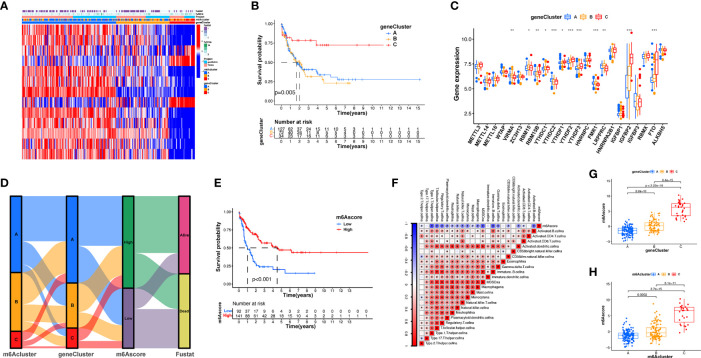
Establishment of m6A signatures. **(A)** Unsupervised cluster of overlapping m6A phenotype-associated genes to categorize patients into diverse genome subgroups, termed m6A genetic clusters A–C. **(B)** KM curves indicating that the m6A modification genome phenotypes are remarkably associated with differing overall survival of 233 patients with AML, of which 127 patients were in genetic cluster A, 72 patients were in genetic cluster B, and 34 patients were in genetic cluster C (*p* < 0.05, log-rank test). **(C)** The expression of 23 m6A modulators in the three genetic clusters. The upper and lower ends of the boxes represent the quartile deviation of the data. The lines in the boxes represent the midvalue, and the black points represent the outliers. The asterisks represent the statistical *p*-value (^*^ *p* < 0.05; ^**^ *p* < 0.01; ^***^ *p* < 0.001). **(D)** Alluvial illustration representing the variation in m6A clusters, genetic clustering, m6A score, and fustat. **(E)** Differences in matrix-stimulated pathways between high m6A score and low m6A score groups. **(F)** Association between m6A score and known genetic markers *via* Spearman assay. Negative association is represented in blue whereas positive association is represented in red. **(G)** Differences in m6A score among the three genetic clusters. A Kruskal-Wallis test was utilized to compare the statistical difference between the three genetic clusters (*p* < 0.001). **(H)** Differences in m6A score among the three m6A modification patterns (*p* < 0.001, Kruskal-Wallis test).

To explore the effects of these m6A modification phenotypes on diverse clinical characteristics and biological behaviors, unsupervised clustering of the AML dataset was performed, and different patterns of m6A modification was found in the cohorts (**Supplementary Figure S4**). An unsupervised cluster analysis was then completed based on the 31 m6A phenotype-associated genes, which was then used to classify patients into different subgroups. Consistent with the cluster group categorization of the m6A modification patterns, the unsupervised cluster algorithm also determined three different m6A modification phenotypes, which we termed m6A gene clusters A–C (**Figure 4A**). This suggests that three different patterns of m6A methylation exist in AML cancers. Of the 233 AML patients, 72 were clustered into the B phenotype, which was associated with a poorer prognosis. In contrast, the patients in cluster C (34 patients) displayed better prognostic outcomes (**Figure 4B**). In these three clusters, obvious differences in m6A regulator expression were discovered, which is consistent with the predicted outcomes of the m6A methylated modification patterns (**Figure 4C**). The obvious differences in m6A gene expression across these three m6A genetic clusters have a nonnegligible regulatory effect on the formation of distinct TME conditions. Nevertheless, these assays are only based on AML patient populations and cannot precisely forecast the m6A methylation modification patterns of individual patients. Given the inhomogeneity and intricacy of m6A modifications, based on these phenotypically associated genes, we established a scoring system to quantify the pattern of m6A modifications in each AML patient, which we termed the m6A score. Alluvial plots were used to visualize the changes in various parameters of a single patient (**Figure 4D**). Genetic cluster A displayed the lowest median and the highest survival rate, while cluster C had the highest median score and increased mortality. The patients with greater m6A scores exhibited significantly improved survival (**Figure 4E**). To better characterize m6A, known characteristics and the m6A score were correlated (**Figure 4F**). The Kruskal-Wallis test showed significant differences in m6A scores among the m6A gene clusters. Genetic cluster A had the lowest median, whereas genetic cluster C displayed the greatest median, suggesting that a low m6A score may be strongly associated with immune stimulation-associated traits, while a high m6A score may be associated with stromal stimulation-associated traits (**Figure 4G**). In addition, m6A score was significantly higher in m6A cluster C and lowest in m6A cluster A (**Figure 4H**). These results suggest that low m6A scores are negatively correlated with immune stimulation, and high m6A scores are also negatively correlated with stromal stimulation. The m6A score can be used to evaluate the m6A modification pattern of individual tumors and further assess the TME infiltrative cell characteristics of tumors.

### Patterns of m6A Modification in TCGA Molecular Subgroups and Cancer Somatic Mutations

**Figure 5:**
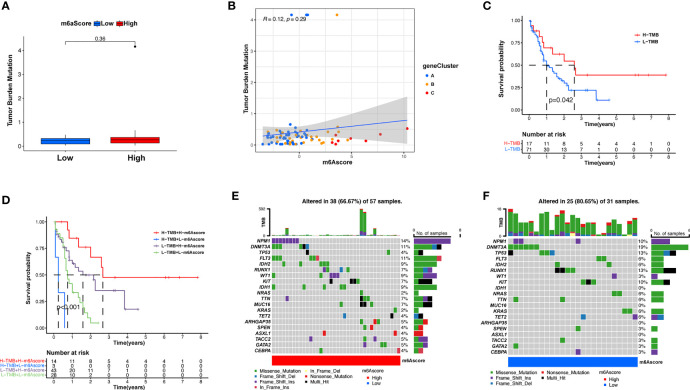
Features of m6A modification in TCGA molecular subgroups and cancer somatic mutation. **(A)** The distribution differences of the tumor mutational burden between low and high m6A score in the TCGA-AML cohort. **(B)** The correlation between m6A score and TMB was not significant. **(C)** Survival assay for low (71 patients) and high (17 patients) TMB groups using KM curves (*p* = 0.042, log-rank test). **(D)** Survival analyses for patients classified by m6A score and TMB using KM curves. H, high; L, Low. (*p* < 0.001, log-rank test). **(E, F)** Waterfall plot of cancer somatic mutations constructed from patients with high m6A score **(E)** and low m6A score **(F)**. Every column represents a single patient. The bar plot above presents TMB, the figure on the right denotes the mutational frequency of each gene. The bar plot on the right represents the level of each variation type.

Subsequently, we determined the m6A score and tumor mutation load and how this was related to patient prognosis. Patients were separated into high or low m6A score groups (**Figure 5A** and **Supplementary Table S7**). m6Ascore and TMB showed a positive correlation (**Figure 5B**). Significantly improved survival existed in the high mutational load group, and there was an even more pronounced survival advantage in patients with high mutational load and high m6A score (**Figures 5C, D**). These results indicate that the m6A score could also be utilized to assess the clinical prognosis of patients. Our team subsequently analyzed the differences in the distribution of certain mutations between the high and low m6A score groups from the TCGA database using the maftools package. As shown in **Figures 5E, F** and **Supplementary Table S8**, the low m6A score group displayed a broader tumor mutational load compared with the high m6A score group, with a ratio of 80.65% and 66.7% mutated genes, respectively. Accumulating evidence suggests that patients with high TMB status exhibit persistent clinical responses to anti-PD-1/PD-L1 immune therapy. Thus, the differences in tumor m6A modification patterns may be key factors mediating clinical reactions to anti-PD-1/PD-L1 immunotherapies. These results will provide new perspectives to explore the mechanisms of m6A methylation patterns in tumor somatic mutations, the formation of TME status, and their role in ICB therapies.

### Verification of Immunomodulator Gene Expression Between AML Samples and Healthy Samples by qRT-PCR and IHC

**Figure 6:**
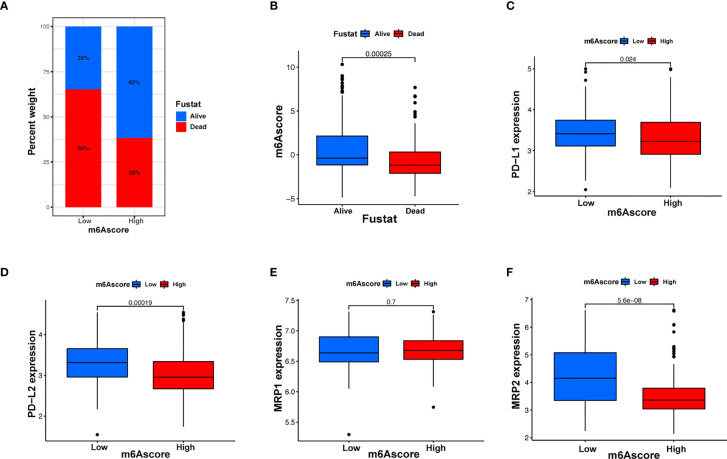
m6A modification patterns and clinical relevance. **(A)** The proportion of patients surviving between the low and high m6A score groups, with a higher proportion surviving in the high scoring group (35% survival in the low m6A score group and 62% survival in the high m6A score groups). **(B)** m6A scores were higher in surviving patients than in patients who died. **(C–F)** Different gene expressions among low and high m6A score groups.

**Figure 7:**
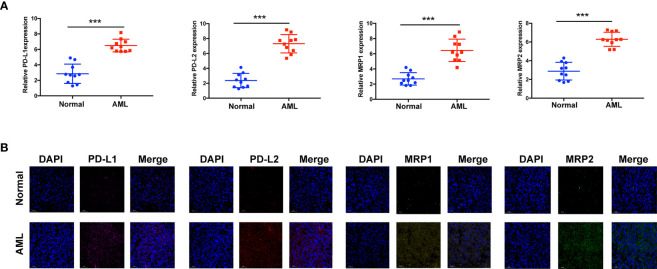
Assay verifying the diversity of immunomodulatory gene expression between healthy and AML samples. **(A)** mRNA expression assay using qRT-RCR. **(B)** Protein expression assay using immunohistochemistry. ***p < 0.001.

Immunotherapy, comprising *PD-L1* and *PD-1* blockade, has become an important breakthrough in tumor treatment. We tested whether m6A modifications could forecast patient reactions to ICB treatment and discovered that patients with high m6A scores demonstrated significant clinical benefits and significantly prolonged survival (**Figures 6A, B**). To further elucidate the correlation between m6A-modified gene expression patterns and host antitumor immune responses, we assessed the expression of immunoregulatory genes in m6A score patients and found that *PD-L1*, *PD-L2*, *MRP1*, and *MRP2* expression was significantly higher in patients with low m6A scores, in contrast to patients with high m6A scores, suggesting a possible response to antitumor immune responses (**Figures 6C–F**). Furthermore, we evaluated the proportion of different genes in the peripheral blood of healthy controls (NC), and patients with AML, identified by qRT-PCR. As shown in **Figure 7A**, compared with the control group, the levels of *PD-L1*, *PD-L2*, *MRP1*, and *MRP2* in the peripheral blood of the patients with AML was significantly increased (*p* < 0.01). Moreover, IHC staining demonstrated the same pattern (**Figure 7B**), suggesting that m6A modifications were crucial for the antitumor immune response in AML. In conclusion, the present research suggests that m6A methylation patterns are closely associated with tumor immunophenotypes and antitumor immune responses, which will help to provide new therapeutic guidance for antitumor immunotherapies.

## Discussion

An increasing number of studies have revealed that m6A modifications have an integral effect on inflammatory events, congenital immunity, and antitumor activity *via* interactions with a variety of m6A modulators. As the majority of studies have focused on one TME cytotype or one modulator, the general TME infiltrative profile under the mediation of the combined action of several m6A modulators is not fully understood. Determining the effect of different m6A modification patterns on TME infiltrative cell activities would help improve knowledge of the TME antitumor immune response and guide more valid immunotherapeutic regimens.

**Figure 8:**
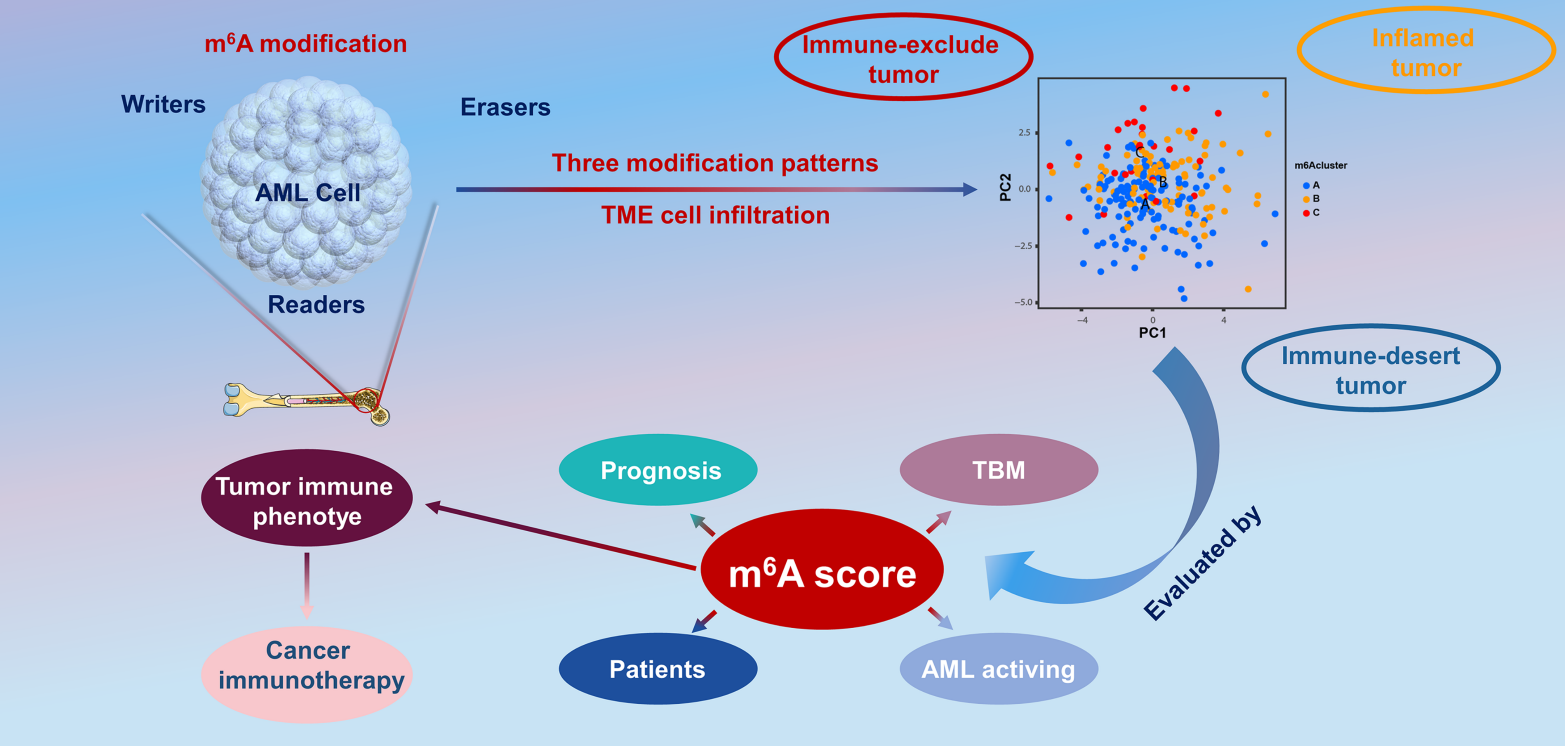
The overview of the dynamic process of m6A RNA methylation modification, which was regulated by “writers,” “erasers,” and “readers” in AML and their potential biological functions.

In the present report, based on 23 m6A modulators, we revealed three prominent m6A methylation patterns characterized by distinctly different TME cell infiltration. Group A was characterized by the stimulation of congenital immunity and matrix, associated with an immunorejection phenotype. Group B was characterized by the stimulation of immunity, associated with immunity metabolism. Group C was characterized by the inhibition of immunity, associated with an immunodeficiency phenotype. In combination with the characteristics of TME infiltrative cells in every cluster, this confirmed that different m6A modification patterns induced by m6A RNA modification is a kinetic and invertible activity under the coordination of a complex of three distinct functional classes of methyltransferases (34), whose roles have been elucidated as “writers” (m6A methyltransferases), “erasers” (m6A demethylases), and “readers” (effectors that recognize m6A) (35, 36). However, there are close interactions between these functional classes, and regulators of the same class can have different effects in different cells (37), which makes it complex and difficult to study the biological functions of m6A modulators in malignant tumorigenesis and the effects on prognosis. Therefore, in the present study, we completed a consensus cluster of m6A modulators based on expression similarity. Clustering is a broadly utilized exploration method for identifying similar groups with coherent properties, which can provide a better understanding of the biology of the global regulation of gene expression and cellular function (38, 39). In this study, we identified three genome subgroups based on m6A signature genes associated with matrix and immune stimulation, which suggested that m6A modifications were important in forming diverse TME conditions. Given the individual heterogeneity of m6A modifications, it is imperative to quantify the pattern of m6A modifications in a single tumor. To this end, we developed a scoring system to assess the m6A modification feature in individual patients with AML, termed the m6A score. Patients with a higher m6A score exhibited improved survival, while those with a lower m6A score displayed a shorter survival time. This suggests that the m6A score is a reliable and powerful tool to comprehensively evaluate the m6A modification patterns of a single tumor, as well as the prognosis of patients (**Figure 8**).

The figures herein show a significant positive association between the m6A score and tumor mutational load. In the present study, we found that m6A methylation patterns have a nonnegligible effect on the formation of diverse stromal and immune TME landscapes, indicating that m6A modifications may influence the potency of ICB. Combined with various biomarkers, including mutational load (40), *PD-L1* expression (41), stromal and immune TME, and MSI status (42, 43), m6A gene characterization may be a more valid forecast method for immune therapies. We also developed the m6A score to assess differences in the expression of the immune prognostic genes *PD-L1*, *PD-L2*, *MRP1*, and *MRP2*. In conclusion, in clinical practice, the m6A score can be utilized to thoroughly assess the m6A methylation patterns of individual patients to further define the immunophenotype of tumors and direct more valid clinical practice. Similarly, the m6A score could be utilized as an independent prognostic biomarker to predict patient survival. Overall, this study provides new insights into cancer immunotherapies that may facilitate the design of new combined medication regimens or fresh immune therapy agents.

## Conclusion

In summary, the present study demonstrates the broad modulatory causal links of m6A methylation patterns on the TME. m6A modification pattern differences are a nonnegligible factor contributing to the inhomogeneity and intricacy of the individual TME. A thorough assessment of individual tumor m6A modification patterns would help improve knowledge of cellular infiltration of the tumor microenvironment and guide more effective immunotherapeutic strategies.

## Data Availability Statement

The datasets presented in this study can be found in online repositories. The names of the repository/repositories and accession number(s) can be found in the article/**Supplementary Material**.

## Ethics Statement

The patient data in this work were acquired from the publicly available datasets of patients whose informed consent were complete. Ethical approval was granted by the Human Research Ethical Board of GPPH.

## Author Contributions

AD and XW designed the study. BJ and JW integrated and analyzed the data. PZ and QZ wrote the manuscript. BJ edited and revised the manuscript. All authors contributed to the article and approved the submitted version.

## Funding

The Fundamental Research Funds for the Central Universities of Central South University under Grant Nos. 2019zzts366 and 2020zzts896 and the guiding project of Qinghai Provincial Health and Family Planning Commission (2018-wjzdx-17).

## Conflict of Interest

The authors declare that the research was conducted in the absence of any commercial or financial relationships that could be construed as a potential conflict of interest.

## Publisher’s Note

All claims expressed in this article are solely those of the authors and do not necessarily represent those of their affiliated organizations, or those of the publisher, the editors and the reviewers. Any product that may be evaluated in this article, or claim that may be made by its manufacturer, is not guaranteed or endorsed by the publisher.

## Supplementary Material

Supplementary Figure 1 Flowchart of AML dataset acquisition and pre-processing.

Supplementary Figure 2 Expression of wild-type WTAP in the m6A regulator.

Supplementary Figure 3 Survival analysis of high and low m6A modification regulators.

Supplementary Figure 4 The different patterns of m6A modification cohorts.

## Abbreviations

TCGA, The Cancer Genome Atlas; TMB, tumor mutational burden; GTEx, genotype-tissue expression; GEO, Gene Expression Omnibus; CNS, central nervous system; ECM, extracellular matrix; GDC, Genomic Data Commons; CNV, copy number variation; ssGSEA, single-sample gene-set enrichment analysis; NKT, natural killer T cell; GO, gene ontology; KEGG, Kyoto Encyclopedia of Genes and Genomes; BP, biological processes; MF, molecular functions; CC, cellular components; PCA, principal component analysis; GPPH, Guizhou Provincial People’s Hospital; KM, Kaplan-Meier method; NK, natural killer cell; MDSCs, myeloid-derived suppressor cells; PDCs, plasmacytoid dendritic cells; ICB, immune checkpoint blockade; OS, overall survival.
