# Supplementary figures and images for: m6A Regulator-Mediated Methylation Modification Patterns and Tumor Microenvironment Infiltration Characterization in Acute Myeloid Leukemia
Source: Front Immunol. 2021 Nov 23;12:789914. doi: 10.3389/fimmu.2021.789914 (PMC8650218; doi:10.3389/fimmu.2021.789914)

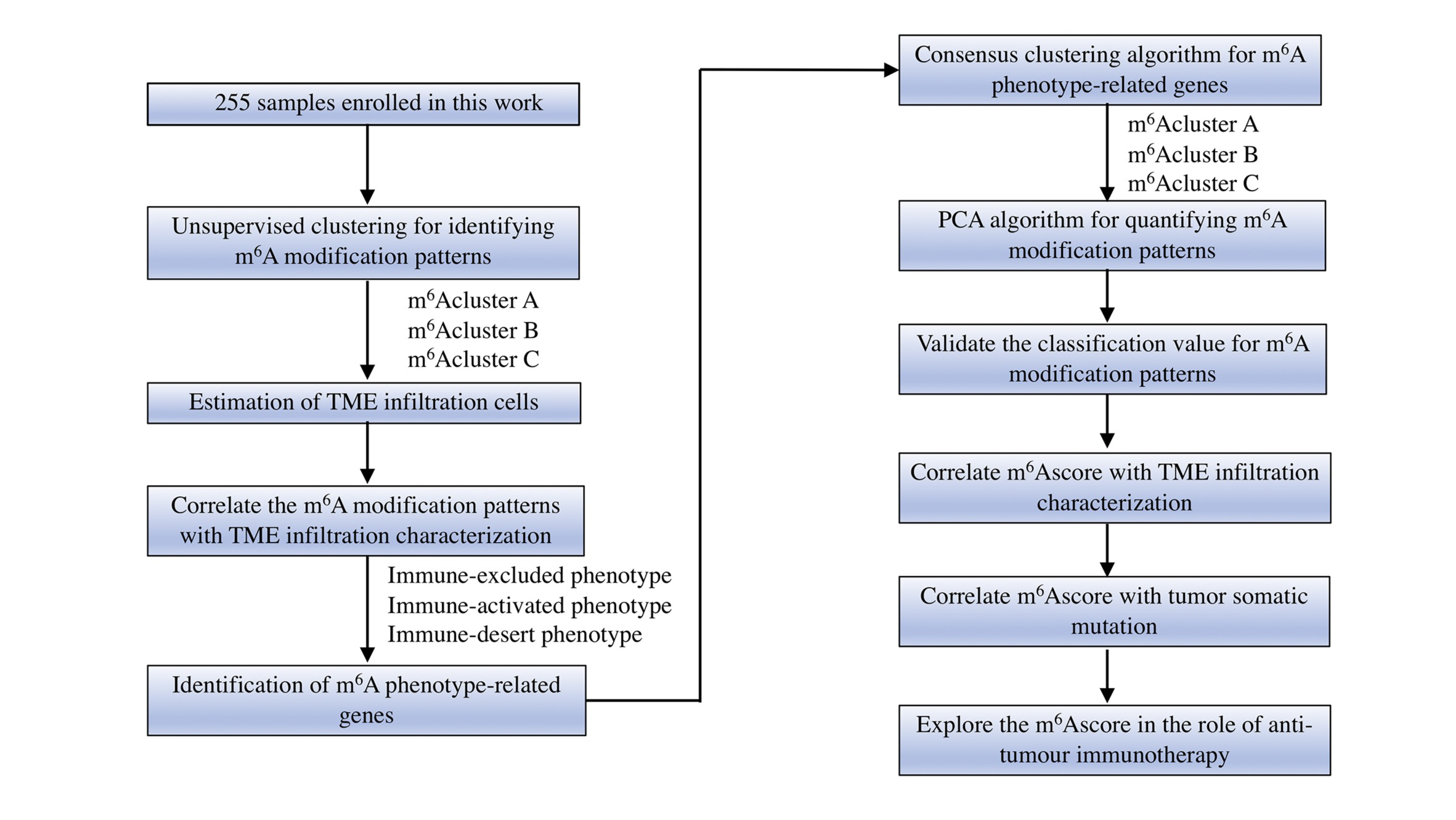

Supplement: Supplementary Figure 1 — Flowchart of AML dataset acquisition and pre-processing. [file Image_1.tif]

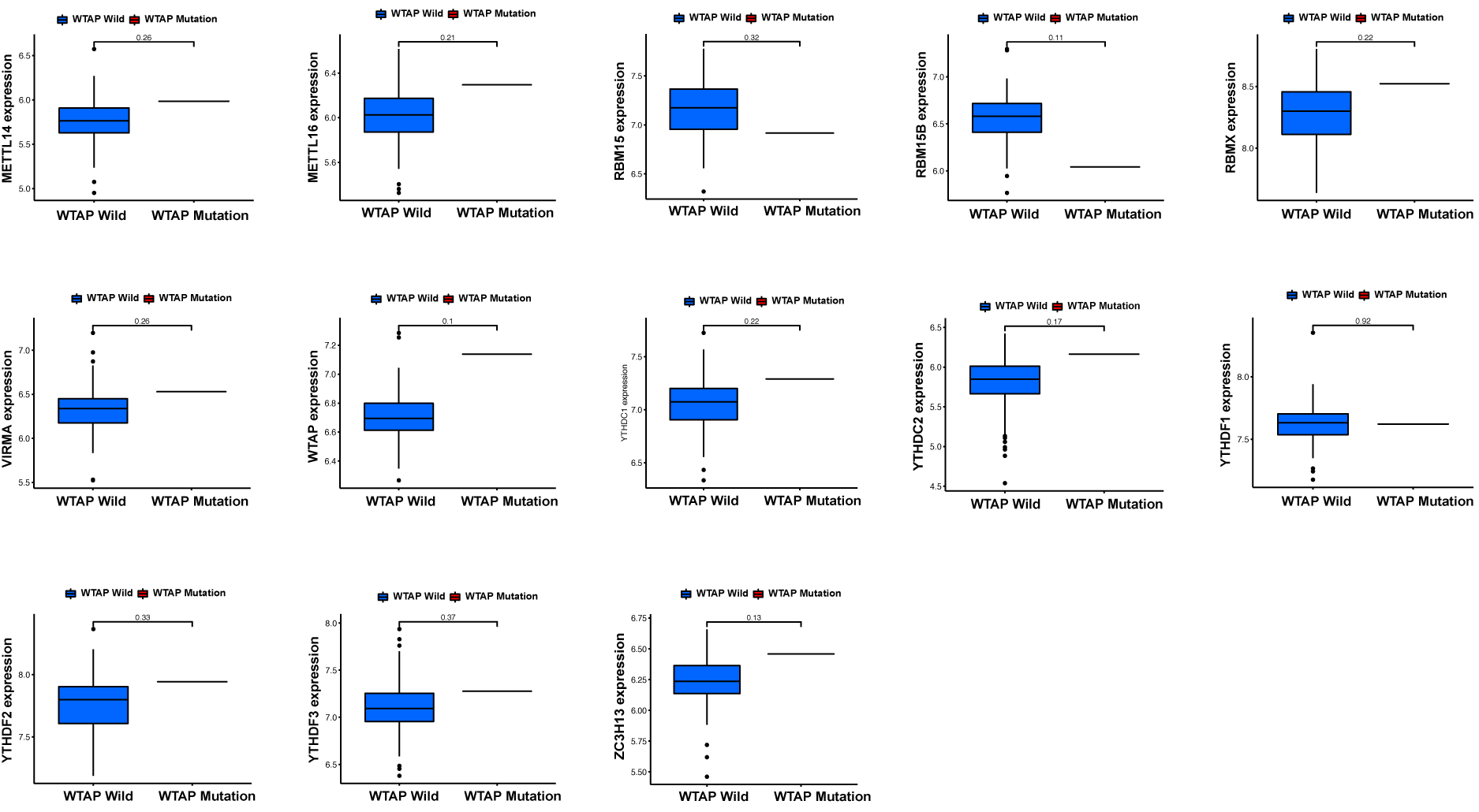

Supplement: Supplementary Figure 2 — Expression of wild-type WTAP in the m6A regulator. [file Image_2.tif]

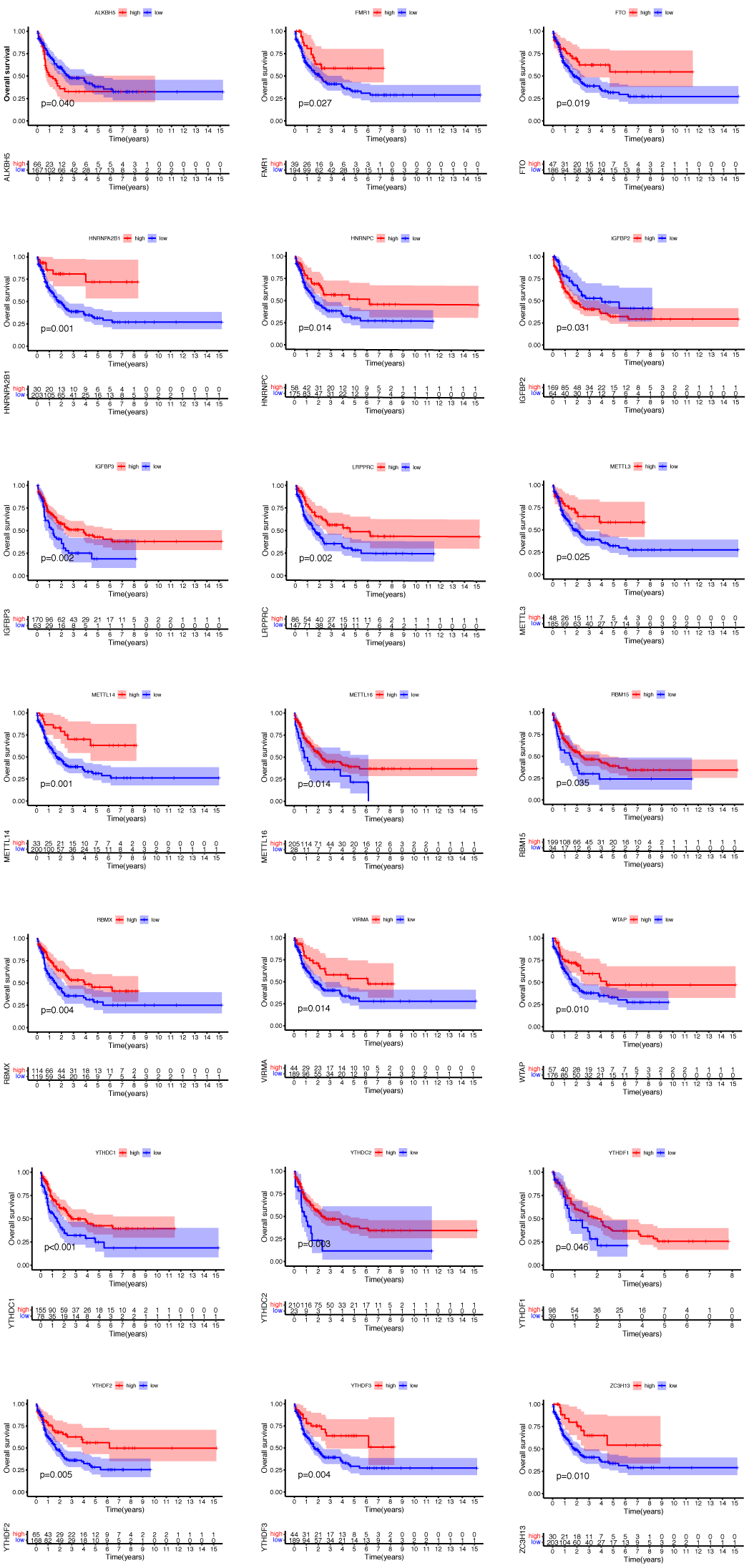

Supplement: Supplementary Figure 3 — Survival analysis of high and low m6A modification regulators. [file Image_3.tif]

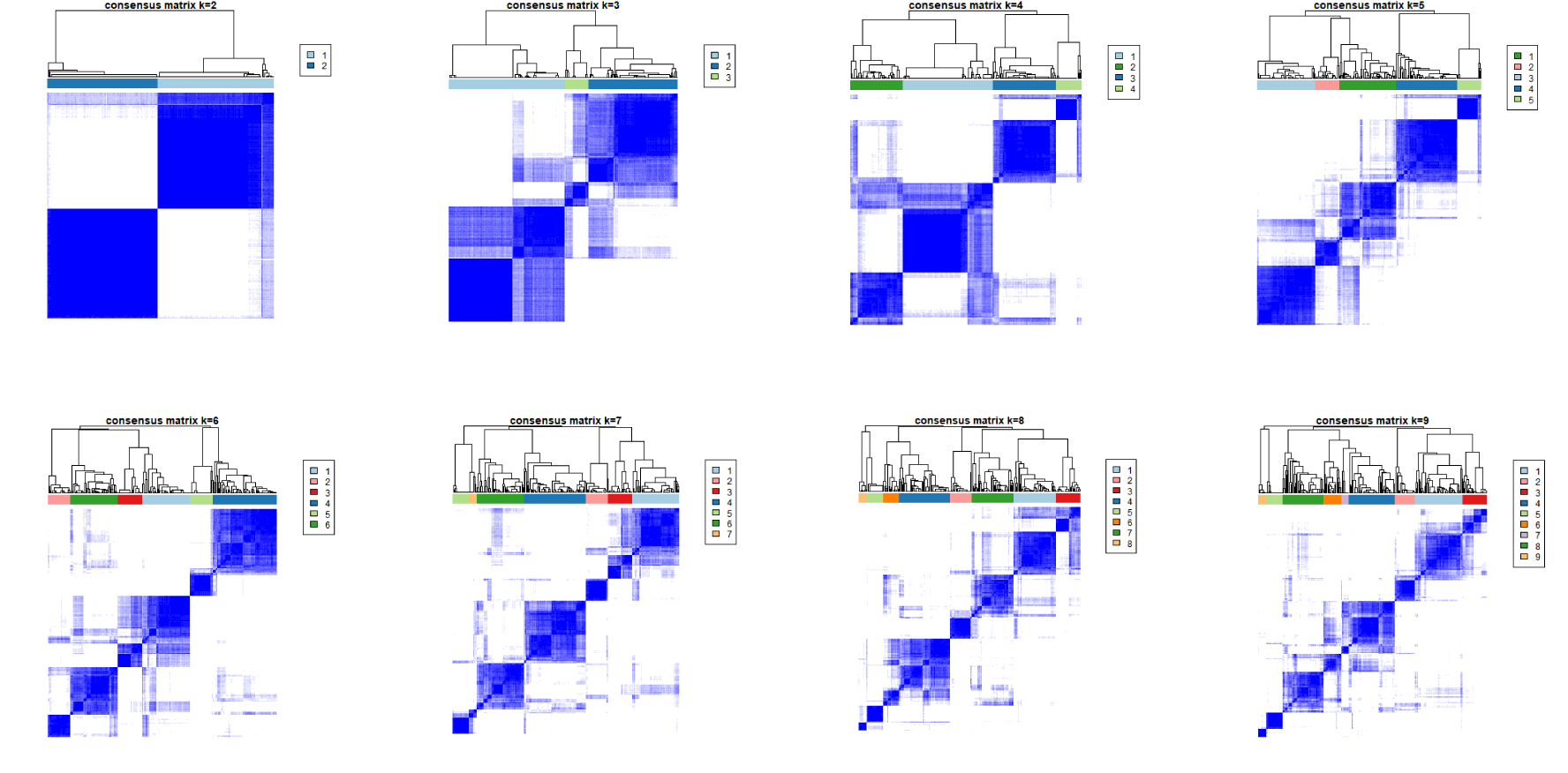

Supplement: Supplementary Figure 4 — The different patterns of m6A modification cohorts. [file Image_4.tif]
